# Arterial Retention of Remnant Lipoproteins Ex Vivo Is Increased in Insulin Resistance Because of Increased Arterial Biglycan and Production of Cholesterol-Rich Atherogenic Particles That Can Be Improved by Ezetimibe in the JCR:LA-*cp* Rat

**DOI:** 10.1161/JAHA.112.003434

**Published:** 2012-10-25

**Authors:** Rabban Mangat, Samantha Warnakula, Faye Borthwick, Zahra Hassanali, Richard R.E. Uwiera, James C. Russell, Christopher I. Cheeseman, Donna F. Vine, Spencer D Proctor

**Affiliations:** 1 Metabolic and Cardiovascular Diseases Laboratory, University of Alberta, Edmonton, Alberta, Canada (R.M., S.W., F.B., Z.H., J.C.R., D.F.V., S.D.P.); 2 Alberta Diabetes Institute, University of Alberta, Edmonton, Alberta, Canada (R.M., S.W., F.B., Z.H., R.R.E.U., J.C.R., D.F.V., S.D.P.); 3 Mazankowski Alberta Heart Institute, University of Alberta, Edmonton, Alberta, Canada (S.D.P.); 4 Department of Physiology, University of Alberta, Edmonton, Alberta, Canada (C.I.C.); 5 Department of Agricultural Food and Nutritional Sciences, University of Alberta, Edmonton, Alberta, Canada (R.M., S.W., F.B., Z.H., R.R.E.U., D.F.V., S.D.P.)

**Keywords:** arterial remodeling, biglycan, metabolic syndrome, triglyceride-rich remnant lipoproteins

## Abstract

**Background:**

Literature supports the “response-to-retention” hypothesis—that during insulin resistance, impaired metabolism of remnant lipoproteins can contribute to accelerated cardiovascular disease progression. We used the JCR:LA-*cp* rat model of metabolic syndrome (MetS) to determine the extent of arterial accumulation of intestinal-derived remnants ex vivo and potential mechanisms that contribute to exacerbated cholesterol deposition in insulin resistance.

**Methods and Results:**

Arteries from control and MetS (insulin-resistant) JCR:LA-*cp* rats were perfused ex vivo with Cy5-labeled remnant lipoproteins, and their arterial retention was quantified by confocal microscopy. Arterial proteoglycans were isolated from control and MetS rats at 6, 12, and 32 weeks of age. There was a significant increase in the arterial retention of remnants and in associated cholesterol accumulation in MetS rats as compared to control rats. Mechanistic studies reveal that increased cholesterol deposition is a result of greater arterial biglycan content; longer glycosaminoglycans and increased production of cholesterol-rich intestinal-derived remnants, as compared to controls. Additionally, perfusion of vessels treated with ezetimibe, alone or in combination with simvastatin, with remnants isolated from the respective treatment group reduced ex vivo arterial retention of remnant-derived cholesterol ex vivo as compared to untreated controls.

**Conclusions:**

Increased progression of atherosclerotic cardiovascular disease in MetS and type 2 diabetes mellitus might be explained in part by an increase in the arterial retention of cholesterol-rich remnants. Furthermore, ezetimibe alone or in combination treatment with simvastatin could be beneficial in ameliorating atherosclerotic cardiovascular disease in insulin resistance and MetS.

## Introduction

Population-based studies have consistently demonstrated that the metabolic syndrome (MetS) is significantly associated with a 1.5- to 3-fold increased risk for atherosclerotic cardiovascular disease (CVD).^1,2,3^ Nevertheless, the mechanisms that accelerate arterial cholesterol deposition during conditions of insulin resistance (IR) and early MetS remain unclear.

The etiology of the “response-to-retention” hypothesis for atherosclerosis suggests that the focal retention of apolipoprotein B (apoB)–containing lipoproteins bound to arterial proteoglycans within the subendothelial matrix of the arterial wall is the key initiating step in atherogenesis.^4,5^ The literature also documents a significant epidemiological association between raised levels of low-density lipoprotein (LDL) cholesterol and CVD risk.^6^ However, the definition of MetS (as defined by the International Diabetes Federation and the American Heart Association) remains independent of LDL cholesterol, suggesting that other important “residual” risk factors could be involved.^7^ Moreover, there is now accumulating evidence that intestinal-derived remnant lipoproteins, including nonfasting postprandial remnants, are significantly elevated in IR (because of overproduction,^8^ impaired clearance,^9,10^ or both) and could promote CVD risk.^11^Furthermore, although it has been shown that as compared to LDL, remnant lipoproteins can contribute physiologically significant quantities of cholesterol to the arterial wall, their relative contributions in IR remain less clear.^12,13^

We know that in the prediabetic phase, chronic IR can lead to either diabetic dyslipoproteinemia (altered lipoprotein composition, increased number of particles, or increased cholesterol per particle) or perturbations in the vessel wall (increased proteoglycans, longer glycosaminoglycans [GAGs], or increased sulfation). Although these factors are thought to influence lipoprotein retention, the comparative contribution of each factor to intestinal-derived remnant lipoprotein retention in vivo remains unclear.

Ezetimibe is a novel pharmaceutical compound that selectively reduces intestinal cholesterol absorption by inhibiting the Niemann-Pick C1–Like 1 (NPC1L1) transporter,^14^ whereas simvastatin is a HMGCoA reductase inhibitor. Some clinical trials have demonstrated that ezetimibe in combination with simvastatin can reduce carotid intima-media thickness,^15^ whereas other trials have shown that addition of ezetimibe to the lipid-lowering regimen does not reduce carotid intima-media thickness.^16^ Still, the effect of these compounds on acute retention of intestinal-derived remnant lipoproteins in IR remains elusive.

In the present study, we used the JCR:LA-*cp* rat model of MetS and IR to (1) determine the extent of arterial accumulation of intestinal-derived remnant lipoproteins ex vivo, (2) assess the effect of IR on the expression and properties of arterial biglycan to bind remnant lipoproteins, (3) understand the contribution of IR to altering remnant particle secretion and composition, and (4) determine the effect of ezetimibe and simvastatin on arterial accumulation of intestinal-derived remnant cholesterol ex vivo.

## Methods

### Animal Model

Male rats of the JCR:LA-*cp* strain were obtained from our breeding colony at the University of Alberta, Edmonton, Canada. JCR:LA-cp rats are homozygous for the corpulent trait (*cp/cp*) are hyperphagic and spontaneously develop IR, which becomes well established by 12 weeks of age.^17^ The corpulent trait has been shown to consist of a nucleotide substitution leading to a stop codon on the extracellular domain of the leptin receptor (ObR).^17^ Furthermore, the *cp/cp* rats show significant postprandial lipemia of apoB48-associated particles, as is observed clinically in humans with IR and MetS. Heterozygous or wild-type rats *(+/cp*) from the same strain are lean and metabolically normal and were used as the controls. For proteoglycan analysis, rats were euthanized at 6, 12, and 32 weeks of age (n=6 at each time point) to represent early, middle, and late IR, and the thoracic aortae were harvested. At 6 weeks of age, lean and MetS rats (n=7 each) received a lipid-balanced diet supplemented with 1% w/w cholesterol to accelerate CVD pathology. Additionally, MetS rats were randomized to a lipid-balanced diet supplemented with either ezetimibe (0.01% w/w) (n=7) or a combination of ezetimibe (0.01% w/w) and simvastatin (0.01% w/w) (n=7) for 8 weeks. At the end of feeding, rats from each group were used for mesenteric duct lymph cannulations (n=4) and arterial perfusion experiments (n=3). Additionally, chow-fed lean (n=8) and IR rats (n=8) were euthanized at 12 weeks of age for enterocyte isolation. Animal care and experimental protocols were conducted in accordance with the Canadian Council on Animal Care and were approved by the University of Alberta Animal Ethics Committee.

At week 7 of treatment and after a 16-hour overnight fast, rats (n=4 from each group) were subjected to an oral fat challenge to assess postprandial apoB48 and cholesterol response.^18^ Plasma total cholesterol concentration was determined with commercially available enzymatic kits (Wako). Plasma apoB48 concentration was quantified by an adapted Western immunoblot method, as previously described.^18^

### Generation of Lymph Chylomicrons (Native TRL) and Remnant Lipoproteins

Rats from each phenotype were fasted for 16 hours to equilibrate gastrointestinal contents. Rats were re-fed 24 hours before lymph cannulation with equal amounts of weighed food (15 g of chow; 3.02 kcal/g) to stimulate basal lymphatic production. The superior mesenteric lymphatic duct was cannulated, and lymph was collected from rats after 4 hours of intragastric saline and glucose (4%) infusion (*fasted* state) and after 4 hours of intragastric intralipid (20%) and glucose (4%) infusion (*fed* state), as described previously.^18^ In 4 hours, intragastric saline and glucose (4%) solution provided 0.96 kcal, and 2% of intralipid (20%) prepared in 4% glucose provided 11.4 kcal. Additionally, for in vivo generation of Cy5-labeled apoB48-containing intestinal-derived remnants (for each group), an intravenous injection of nascent chylomicrons obtained after intragastric intralipid (20%) and glucose (4%) infusion was given to hepatectomized rabbits. Cy5 label was prepared as per manufacturer's instructions. Cy5 label is attached to apoB and has been demonstrated to not transfer between other lipoproteins.^19^ Labeled chylomicron remnants were collected from the plasma after density centrifugation, as described previously.^19^

### Ex Vivo Perfusion of Labeled Remnants

Isolated carotid vessels from rats (n=3 from each group, incorporating both carotids of each rat) were exposed to Cy5-labeled remnant lipoproteins via ex vivo arterial perfusion using an autologous and a cross-over study design. Carotid vessels were perfused under physiological conditions of temperature and pressure (70 mm Hg) with the use of a Harvard Apparatus model UNIPER UP-100, as previously described,^19^ and were standardized to particle number (apoB, 100 μg/mL) for all experiments. Image quantification was done as previously described.^19^

### Image Quantification

Fluorescent lipoproteins were visualized by the Zeiss 510 confocal laser (Kr/Ar) scanning microscope or Perkin Elmer spinning disk microscope, and images were viewed by Zeiss LSM Image Browser Version 4.2.0.121 or Volocity, as previously described.^19^ The fluorescent intensity associated with remnants was determined by digital analysis in 3 dimensions with NIH ImageJ software (version 1.36b) and the plug-in LSM reader. The biochemical profile for each remnant lipoprotein preparation was determined before each experiment. Direct enzymatic colorimetric assays were used to measure triglyceride and total cholesterol.^19^ ApoB protein from remnants was quantified by an adapted Western immunoblot technique, and cholesterol in the perfusate was quantified by colorimetric assay.^18,19^ The fluorescent intensity of the perfusate per unit volume was also collected for every experiment, under identical conditions, in Z-series. These data (intensity and the cholesterol concentration of the perfusate) subsequently were used to calculate the corresponding mass of cholesterol retained, as previously described.^19^

### Enterocyte Isolation

Approximately 30 cm of jejunal tissue was excised and used for enterocyte isolation by the Weiser method.^20^ Briefly, enterocytes were detached by serial incubations with dithiothreitol and citrate with shaking.^20^ The removal of calcium by EDTA and citrate results in a loss of protein-mediated calcium-dependent adhesion of cells to the basement membrane. Enterocyte fractions (n=10) were collected sequentially from 1 through 10, with fraction 1 representing the proximal (tip) and fraction 10 representing the distal (crypt) portion of the intestinal villus. Isolated cell fractions were centrifuged at 1000 rpm for 2 minutes, and the supernatant was discarded. Pellets containing enterocytes were washed in phosphate-buffered saline twice and finally resuspended in 1 mL of phosphate-buffered saline. Cell count was performed on fresh cells from each fraction with a hemacytometer. Additionally, cell-associated protein concentration was determined on lysed cells. The concentration of apoB48 was determined by an adapted Western immunoblot method, as previously described.^18,19^

### Enterocyte Morphology

Intestinal sections were excised from lean and MetS rats after a 16-hour overnight fast. The length of the intestine was flushed with phosphate-buffered saline and was measured. A 1-cm jejunal portion of the intestine was cut, inverted, and sliced transversely. Sliced jejunal sections were oriented in Optimal Cutting Temperature medium, preserved by snap-freezing in liquid nitrogen, and kept at −80°C for histological analysis. Histological characteristics of intestinal villi were assessed in tissue cryosectioned (8 μm thick) and stained with hematoxylin and eosin dyes. Morphological observations of the intestinal lumen (villus length, width, and area) were quantified with Axiovision software 7.0.

### Extraction of Small Leucine-Rich Proteoglycans From Rat Aortae

Proteoglycans were extracted from aortae of rats of differing ages (as described above) by using the 4 mol/L GnHCl-containing extraction solution, as previously described.^21^ The proteoglycan extract was concentrated further by eluting on a diethylaminoethyl column and pooling 1,9-dimethylmethylene blue–positive fractions.^21^ The final pooled fraction was digested with chondroitin ABC lyase (Sigma) and separated on a 4% to 12% Bis-Tris gel. Western blots were probed by LF106 antibody (kind gift from Dr Larry Fischer, National Institutes of Health, Bethesda, MD) for murine biglycan and by 6D6 antibody (kind gift from Dr Paul Scott, University of Alberta, Canada) for decorin. For GAG analysis, pooled proteoglycan samples (intact as well as digested by chondroitin ABC lyase) were separated on a 4% to 12% Bis-Tris gel that was stained with dimethylmethylene blue, a cationic dye that binds to the sulfated GAGs.

### Recombinant Biglycan

For the production of human recombinant biglycan, cDNA (Origene) was used to transfect host FS293 cells, as described elsewhere.^12^ The culture medium was semipurified by diethylaminoethyl Sephacel column. The dimethylmethylene blue–positive fractions were pooled and dialyzed. GAG chains were characterized on a dimethylmethylene blue–stained gel, and the size of protein core was validated after ABC lyase digestion followed by a Western blot.

### Solid-Phase Binding Assay

Recombinant human biglycan was diluted in HEPES-buffered saline, added (2 μg/well) to a Maxisorp immunoplate (Nunc), and incubated at room temperature overnight to allow adherence, as per Boren et al.^12,22^ Before the addition of lipoproteins, all wells were washed with HEPES-buffered saline. The number of lipoproteins in each well was standardized to a range of apoB concentrations (0.625, 1.25, 2.5, 5, 7.5, 10, and 20 μg/mL) to allow for generation of binding affinity (K_D_) and binding capacity (B_max_) values. Nonspecific binding sites were blocked with HEPES-buffered saline+1% bovine serum albumin. Bound lipoproteins were rinsed 3 times with HEPES-buffered saline+0.02% Tween. To detect bound lipoproteins, horseradish peroxidase–conjugated anti–human/rodent apoB (R&D BioDesign) diluted (1/750) in HEPES-buffered saline–Tween+0.1% bovine serum albumin was used. Finally, 1-Step Turbo TMB-ELISA (Pierce) was used to allow for color development, and absorbance was measured at 450 nm.

### Heart Histology and Myocardial Lesion Frequency

After 8 weeks of treatment, hearts from all groups (n=4 per group) were fixed in formalin, embedded in a single paraffin block, sectioned, and then stained with hematoxylin and eosin, as previously described.^23^ Heart sections were examined blindly by an independent experienced observer, and the number of ischemic lesions was identified in each of the sections, as reported elsewhere.^23^ Myocardial lesions were categorized as Stage 1 through Stage 4, as previously described^23^: Stage 1, area of early necrosis with no long-term inflammatory cell infiltration; Stage 2, area of cell lysis with chronic inflammatory cell infiltration; Stage 3, nodules of chronic inflammatory cell infiltration; and Stage 4, old scarred lesions.^23^ The number of lesions determined from the sections of individual hearts was aggregated, and the mean incidence for each group was calculated.

### Statistical Analysis

Graphpad Prism was used for statistical analysis. Unpaired *t* test and 1-way analysis of variance (ANOVA) with Tukey's post hoc analysis were used. Nonlinear regression analysis and “1-site binding” equation were used to generate binding curves and B_max_ and K_D_ values. Correlation between fasting insulin values and arterial biglycan mass was determined by linear regression.

## Results

### Ex Vivo Retention of Labeled Remnants in Carotid Vessels of JCR:LA-*cp* Rats

**Figure 1.**
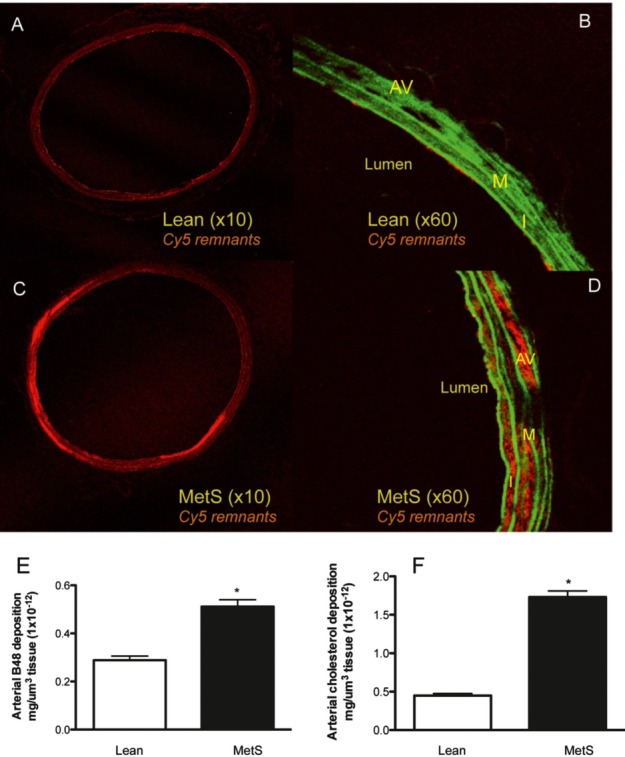
Ex vivo retention of Cy5-labeled remnant lipoproteins and associated cholesterol in carotid arteries of the lean and MetS JCR:LA-*cp* rats after autologous perfusion (A through F). For autologous perfusion, MetS rat carotid vessels were perfused with remnants obtained from MetS rats, and lean carotid vessels were perfused with remnants obtained from lean rats. Representative images of the retention of Cy5-labeled remnants in carotid vessels of lean (A and C, ×10 magnification) and MetS (B and D, ×60 magnification) rats. Morphology of the vessel is shown in green and Cy5-labeled remnants in red. I indicates intima; M, media; AV, adventitia; and MetS, metabolic syndrome. Ex vivo retention of remnants in carotid vessels of lean and MetS rats was quantified further by measuring the fluorescent intensity in the linear range and calculating the corresponding number of apoB particles (using known concentration of apoB in the perfusate) (E) as well as the corresponding concentration of cholesterol (F), for each remnant preparation. *P<0.0001 (n=3, incorporating both carotids, 2 to 3 sections from each vessel, complete cross sections at ×10 magnification from each rat). (The unit on the y-axis in E and F also can be expressed as femtograms of apoB48). Images were collected in 3 dimensions for autologous perfusion (A through F for each arterial section).

In autologous perfusion experiments (carotid vessels isolated from MetS rats perfused with remnants isolated from MetS rats), we observed 76% greater retention of remnant lipoproteins (number of particles) in carotid vessels from MetS rats (0.51±0.02 ×10^−12^ mg/µm^3^ tissue) relative to controls (0.29±0.01 ×10^−12^ mg/µm^3^ tissue) (Figure 1A through 1E). In addition, there was a 2.5-fold increase in the accumulation of remnant-derived cholesterol in arteries from IR rats (1.73±0.08 ×10^−12^ mg/µm^3^) compared to control (0.45±0.02 ×10^−12^ mg/µm^3^, *P*<0.001) (Figure 1F).

Here, we would like to mention that it is usually assumed that apoB lipoproteins that enter across the endothelium would also leave across the endothelium. However, we found that the labeled lipoprotein particles egress from the subendothelium via the media and adventitia, thereby explaining the presence of fluorescent label in all the layers (Figure 1A through 1D).

**Figure 2.**
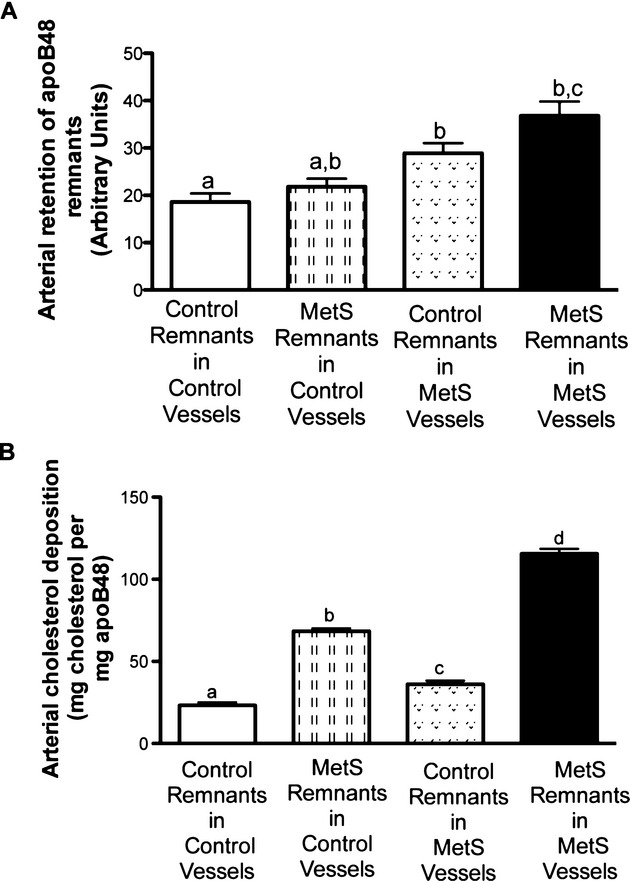
Ex vivo retention of Cy5-labeled remnant lipoproteins and associated cholesterol in carotid arteries of the lean and MetS JCR:LA-*cp* rats after crossover perfusion (A and B). For crossover perfusion, lean remnants were perfused into lean and MetS carotid vessels and vice versa. Ex vivo retention of remnants in carotid vessels of lean and MetS rats was quantified further by measuring the fluorescent intensity in the linear range and calculating the corresponding number of apoB particles (using known concentration of apoB in the perfusate) (A) as well as the corresponding concentration of cholesterol (B) for each remnant preparation. Different letter subscripts (a through d) all indicate a statistical significant difference of *P*<0.05 between the groups according to 1-way ANOVA (n=3, incorporating both carotids, 2 to 3 sections from each vessel, complete cross sections at ×10 magnification from each rat). MetS indicates metabolic syndrome. Values with the same letter subscript are not statistically different. Images were collected in 2 dimensions for crossover perfusion experiments for each arterial section.

**Figure 3.**
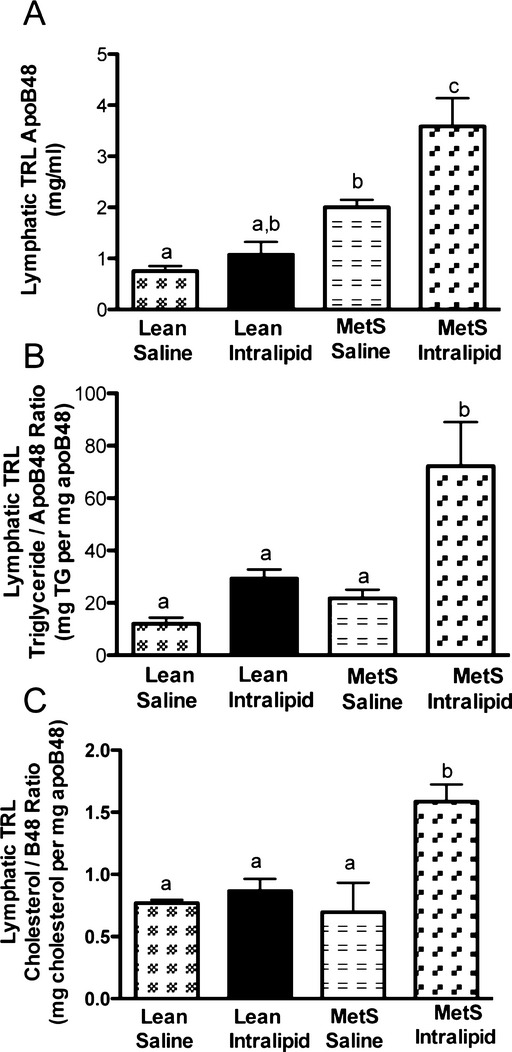
Lymphatic apoB48 secretion (A), triglyceride/apoB48 ratio (B), and cholesterol/apoB48 ratio (C). Lymph apoB48 (A), triglyceride/apoB48 (particle size) (B), and cholesterol/apoB48 (cholesterol per particle) (C) were measured in native TRL after intragastric saline and glucose (4%) infusion (fasted state) or intragastric intralipid (20%) and glucose (4%) infusion (fed state) in the lean and MetS JCR:LA-*cp* rats. Different letter subscripts (a through c) all indicate a significant difference of *P*<0.05 between the groups according to 1-way ANOVA (n=4). MetS indicates metabolic syndrome. Values with the same letter subscript are not statistically different.

To determine the differential contribution of the lipoproteins and the vessel wall to exacerbating intestinal-derived remnant retention in IR, we performed crossover perfusion experiments. Interestingly, there was no difference in the retention (particle number) of remnant lipoproteins isolated from MetS rats when perfused into vessels from control rats as compared to the retention of remnants isolated from control rats perfused into vessels from control rats (Figure 2A). However, there was significantly greater (1.5-fold) arterial retention (particle number) of remnants isolated from control rats and perfused into vessels from MetS rats (28.9±2.12 arbitrary units) as compared to the retention of remnants isolated from control rats and perfused into vessels from control rats (18.6±1.79 arbitrary units) (Figure 2A). Furthermore, there was significantly more cholesterol deposition from remnants isolated from MetS rats (68.45±1.7 mg cholesterol per 1 mg apoB48) than from remnants isolated from control rats (23.25±1.79 mg cholesterol per 1 mg apoB48) (Figure 2B). To appreciate the contribution of particle composition to increased cholesterol deposition, lymph cannulation experiments revealed that MetS rats have overproduction of large intestinal-derived particles (Figure 3A and 3B) and are cholesterol rich (Figure 3C). It is also noteworthy that lymphatic triglyceride-rich lipoprotein (TRL) particles from IR rats remain cholesterol rich even after significant hydrolysis (and exchange of cholesterol esters and triglycerides with other lipoproteins) to smaller, denser remnant particles (Table 1).

**Table 1.** Biochemical Characteristics of Intestinal-Derived Remnants

Lipoproteins	Cholesterol, mg/mL	ApoB, mg/mL	Cholesterol/apoB
Lean remnants	0.62	0.398	1.55
MetS remnants	0.75	0.209	3.50

### Particle Characteristics of Lymphatic TRL During Fasted and Fed States

To investigate the mechanism for the production of cholesterol-rich remnants in MetS, we determined particle characteristics of native TRL. There was a significant increase in the apoB48 concentration (particle number) in the TRL obtained from MetS rats in both fasted (intragastric saline and glucose infusion) and fed (intragastric intralipid and glucose infusion) states (Figure 3A). However, particle size (TG/B48 ratio) and cholesterol per particle were significantly increased in the TRL obtained from MetS rats as compared to lean rats in the fed state only (Figure 3B and 3C).

**Figure 4.**
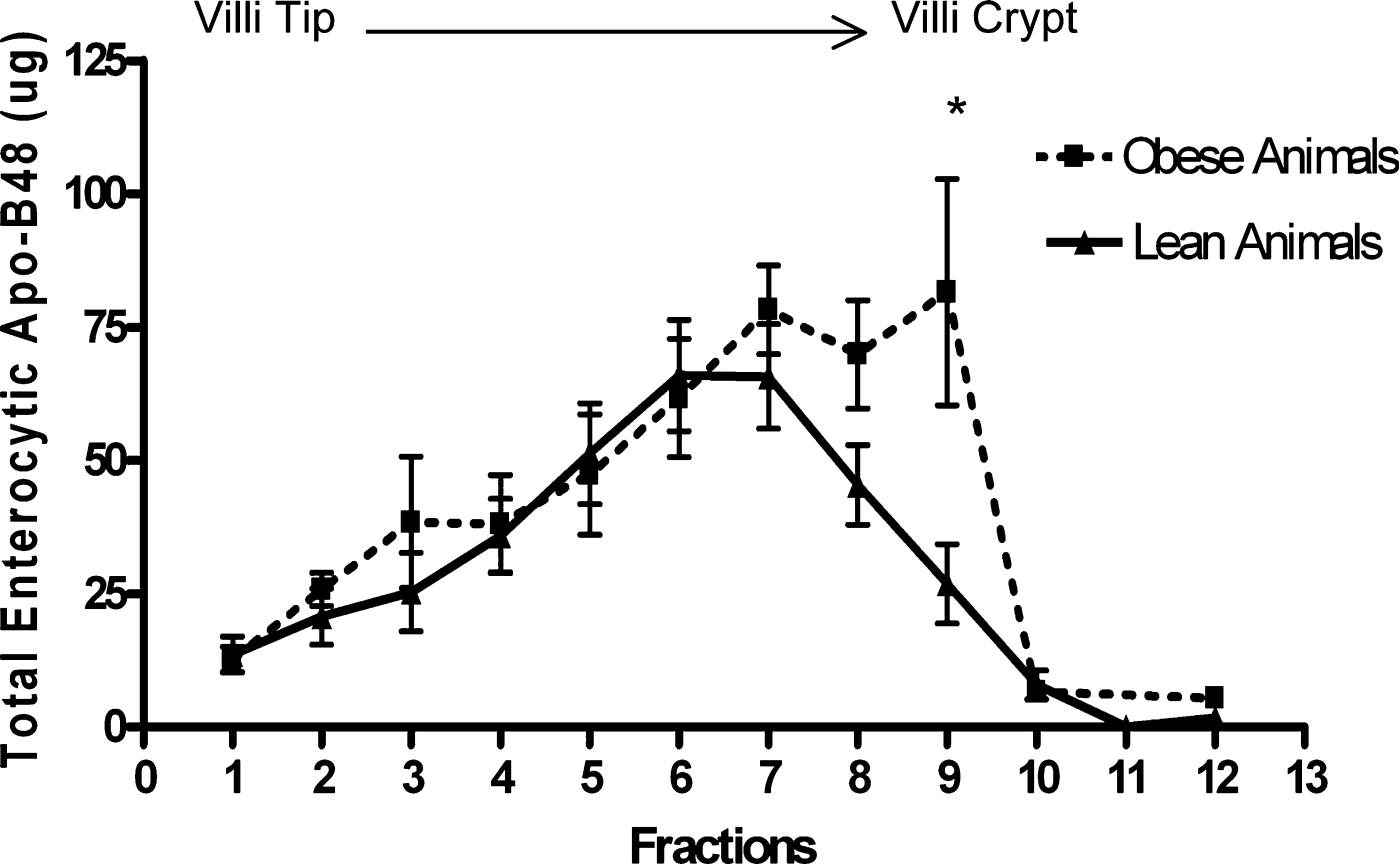
Cell-associated apoB48 mass along the length of the intestinal villus. Approximately 30 cm of jejunal tissue was excised and used for enterocyte isolation by the Weiser method (see details in Methods). ApoB48 mass was determined for each isolated cell fraction via immunoblotting. MetS indicates metabolic syndrome. *P<0.05 represents statistical significance in apoB48 from isolated cell fractions between phenotypically lean (n=8) and MetS (insulin-resistant) (n=8) rats.

**Figure 5.**
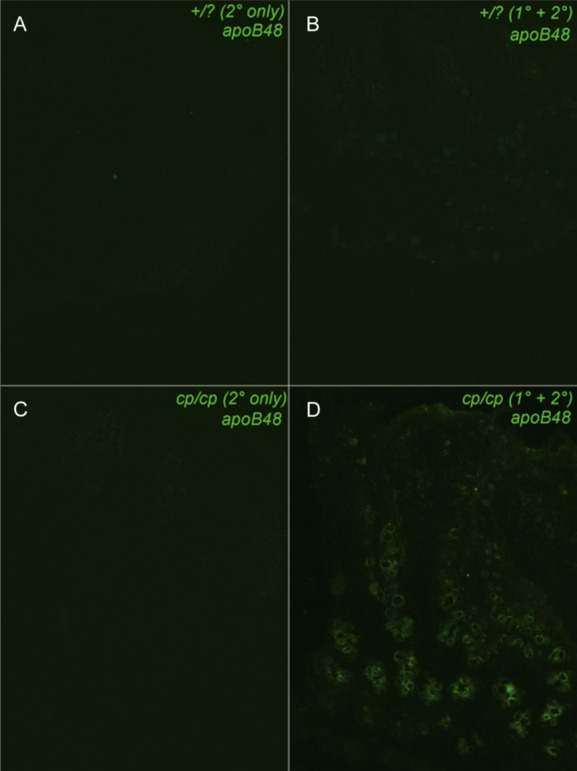
Immunohistochemical visual of the distribution of ApoB48 along the intestinal villus. A and B, Intestinal sections from lean animals (+/?) using secondary antibody alone or primary and secondary antibody, respectively. C and D, Sections from a MetS (*cp/cp*) rat treated with secondary antibody alone or primary and secondary antibody, respectively. MetS indicates metabolic syndrome. Images were captured at 20× magnification.

Investigation of the intestine from MetS rats revealed intestinal hypertrophy, as determined by increased jejunum weight and length and intestinal villi height and volume, relative to lean rats (Table 2 and Figure 4). In addition to the increase in number of enterocytes observed in the small intestine of MetS rats, an abundance of apoB48 also was found at the crypt of the villi (Figure 5).

**Table 2.** Characterization of the Intestinal Morphology of Lean and MetS JCR:LA*-cp* Rats

	Lean Rats (+/?)	IR (*cp/cp*)
Weight, kg	432±17.7^a^	750±10.4^b^
Insulin, mU/L	12.4±1.6^a^	423.5±163.4^b^
Length of intestine, cm	94±9.8^a^	111±6.8^b^
Weight of jejunum, g	3.02±0.23^a^	4.68±0.72^b^
Villus length, μm	499.8±17.75^a^	631.9±28.84^b^
Villus width, μm	193.72±16.66	190.3±9.36
Villus area, μm^2^	79530±8532^a^	106500±8668^b^

Data represent the phenotypic characteristics of Lean and MetS rats.

Different letter superscripts indicate significance *P*<0.05 as analysed by unpaired *t* test vs. lean rats (n=8 lean and n=8 IR).

### Binding of Native Lymphatic TRL Isolated From MetS Rats to Human Biglycan During the Fasted and Fed States

To assess the effect of postprandial dyslipidemia on the direct binding efficacy of remnant particles to atherogenic proteoglycans, native lymphatic TRL were isolated from control and MetS rats during the fasted (intragastric saline and glucose infusion) and fed (intragastric intralipid and glucose infusion) states. We did not observe any difference in the K_D_ to biglycan for lipoproteins collected from control or fasted MetS rats (data not shown). In contrast, the TRL collected from MetS rats during the fed state was shown to have 1.26-fold higher B_max_ to biglycan (0.215±0.009 absorbance units) (Table 3) than that of TRL collected from lean rats during the fed state (0.160±0.009 absorbance units, *P*<0.01).

**Table 3.** Binding of Native TRL Collected From Lean and IR JCR:LA-*cp* Rats Under Fasted and Fed Conditions to Recombinant Human Biglycan

Treatment	B_max_±SEM (Absorbance Units)	K_D_±SEM	Cholesterol per Particle (Milligrams Cholesterol per Milligram apoB48)
Lean saline	0.1182±0.01^a^	0.2193±0.32^a^	0.66
Lean intralipid	0.1480±0.008^b^	0.1769±0.17^a^	0.83
MetS saline	0.1605±0.008^a,b^	0.1855±0.11^a^	0.37
MetS intralipid	0.2150±0.009^c^	0.1058±0.09^a^	1.37

Please see Methods for description of the solid-phase binding assay used to generate B_max_ values. Rats from each phenotype were fasted for 16 hours to equilibrate gastrointestinal contents. Rats were re-fed 24 hours before lymph cannulation with equal amounts of weighed food to stimulate basal lymphatic production. The superior mesenteric lymphatic duct was cannulated and lymph collected from MetS and control rats after intragastric saline and glucose (4%) infusion (fasted state) and after intragastric intralipid (20%) and glucose (4%) infusion (fed state). Cholesterol and triglyceride were measured by colorimetric assay with a kit (WAKO). SEM indicates standard error of the mean. Different letter subscripts (a through c) all indicate a significant difference of *P*<0.05 between the groups using 1-way ANOVA. Values with the same letter subscript are not statistically different.

**Figure 6.**
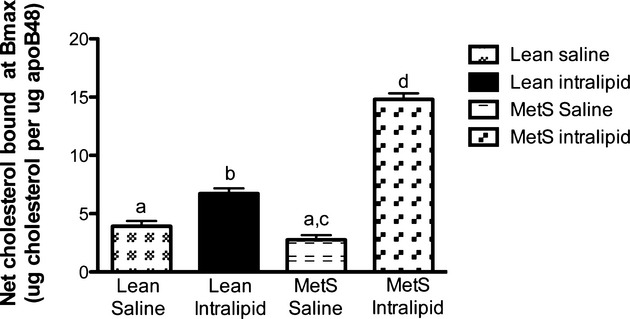
Net cholesterol bound to biglycan (associated with native TRL) at B_max_ as assessed from the solid-phase binding assay. Native TRL were isolated after intragastric saline and glucose (4%) infusion (fasted state) or intragastric intralipid (20%) and glucose (4%) infusion (fed state) in the lean and MetS JCR:LA-*cp* rats. B_max_ values (maximal binding capacity) were calculated from a solid-phase binding assay, which was standardized to a range of apoB48 concentrations to allow for generation of K_D_ and B_max_ values. For B_max_ values, see Table 3. Net cholesterol bound was calculated as the product of the B_max_ value for the respective TRL fraction and its cholesterol concentration. Different letter subscripts (a through d) all indicate a significant difference of *P*<0.05 between the groups according to 1-way ANOVA (n=3). MetS indicates metabolic syndrome. Values with the same letter subscript are not statistically different.

We further calculated the mass of bound cholesterol at the corresponding B_max_ for each of the solid-phase binding assay experiments. Notably, native TRL-derived bound cholesterol was significantly higher for particles isolated during the fed state regardless of phenotype (4.32±1 versus 6.72±0.9 mg cholesterol per 1 mg apoB48 for lean rats in the fasted and fed states, respectively, *P*<0.05; 2.75±0.8 versus 14.8±0.9 mg cholesterol per 1 mg apoB48 for MetS rats in the fasted and fed states, respectively, *P*<0.01) (Figure 6).

It was also interesting to note that the mass of cholesterol bound to biglycan associated with TRL from MetS rats collected during the fed state was double that for cholesterol associated with particles from non-IR rats (Figure 6).

### Effect of MetS on the Remodeling of Aortic Biglycan, Decorin Protein Core, and Sulfated GAGs

**Figure 7.**
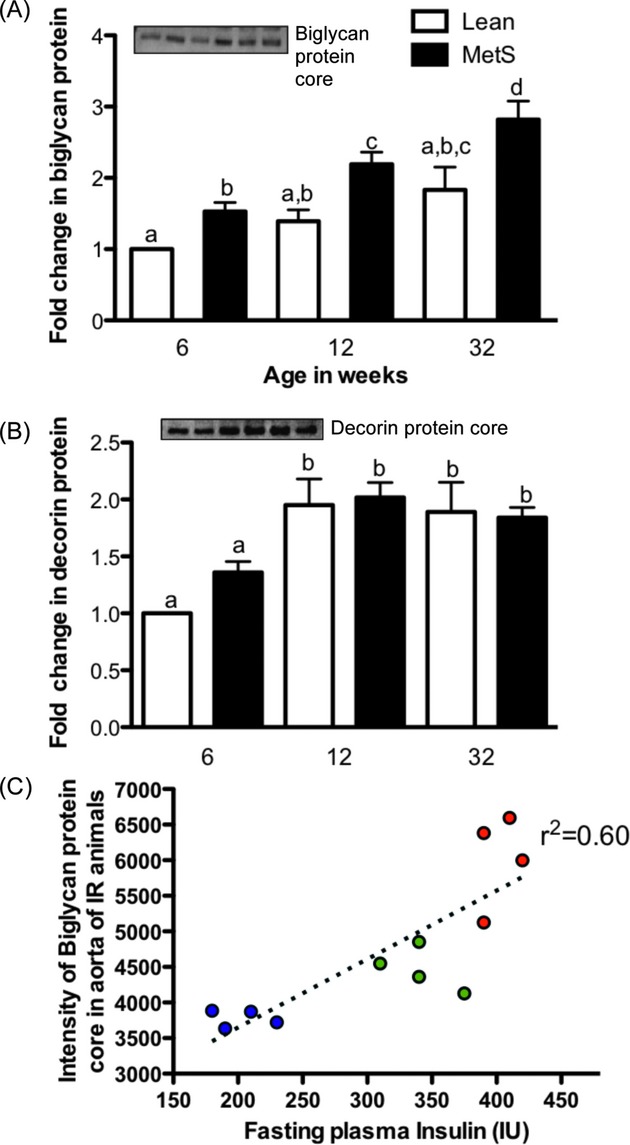
Fold change in biglycan core protein content (A) and decorin protein core content (B) and correlation of biglycan core protein with fasting insulin concentration in the MetS rats (C). Immunoblot shows arterial biglycan core protein (A) and decorin protein core (B) after extraction of total arterial proteoglycans and enzymatic digestion with chondroitin ABC lyase. Pooled rat samples of JCR:LA-*cp* rats of increasing age (n=3; 2 to 3 rats each) were used for proteoglycan extraction. Graph shows fold differences compared to lean phenotype at 6 weeks of age. Different letter subscripts (a through d) all indicate a significant difference of *P*<0.05 between the groups according to 1-way ANOVA. MetS indicates metabolic syndrome. Values with the same letter subscript are not statistically different. Biglycan core protein with increasing age in MetS rats was correlated with fasting insulin concentration in MetS rats with increasing age (C). Blue, green, and red dots on the graph represent 6, 12, and 32 weeks of age, respectively.

We measured the expression pattern of arterial proteoglycans in JCR:LA*-cp* rats in vivo by assessing the abundance of biglycan and decorin in aorta. The fold difference of biglycan protein mass extracted from aorta of MetS rats was significantly increased with age (1.53±0.12-fold, 2.19±0.17-fold, and 2.83±0.26-fold at 6, 12, and 32 weeks, respectively; *P*<0.05) (Figure 7A). In contrast, the mass of decorin protein extracted from the aorta did not differ between the phenotypes at any time point (Figure 7B). Decorin protein core isolated from rats at 12 and 32 weeks of age was significantly greater (*P*<0.05) than at 6 weeks of age (Figure 7B). Interestingly, aortic biglycan protein core isolated from MetS rats of increasing age had a significant correlation (*r*^*2*^=0.60, *P*=0.003) with fasting plasma insulin concentration (Figure 7C).

**Figure 8.**
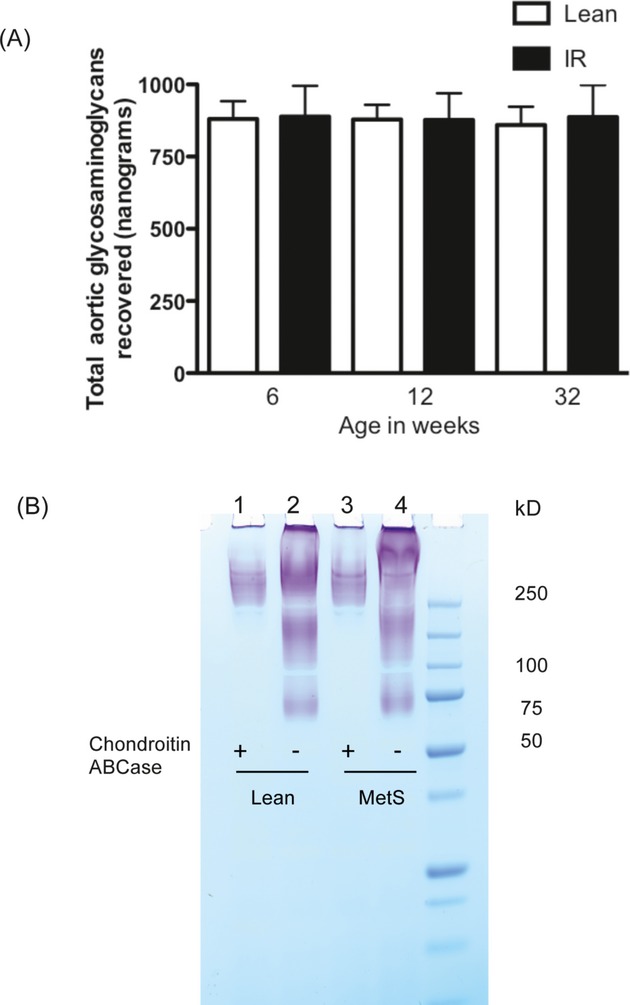
Total aortic sulfated proteoglycans (A) and characterization of total sulfated GAGs isolated from lean and MetS rats (B). Total sulfated GAGs were isolated from lean and MetS JCR:LA-*cp* rat aortae at 12 weeks of age. Intact and enzymatically digested (chondroitin ABC lyase) samples were characterized on a SDS-PAGE gel by staining the gel with 1,9-dimethyl methyleneblue (DMMB) dye, which stains the sulfated groups. DMMB-stained gel (B) shows GAGs isolated from lean (lane 2) and MetS (lane 4) JCR:LA-*cp* rat aortae. Lanes (1) and (3) show chondroitin ABC lyase–digested samples of lean and MetS total sulfated GAGs, respectively. MetS indicates metabolic syndrome.

We also noted that there was no change in total aortic sulfated GAGs from rats of any age or between phenotypes (Figure 8A). Nevertheless, at 12 weeks of age, chondroitin ABC lyase–digestible GAGs showed heavier (longer) GAG chains from arteries derived from MetS rats versus controls (Figure 8B). No difference was observed in the chondroitin ABC lyase–resistant (heparan sulphate) GAG chains with this model.

### Effect of Ezetimibe and Simvastatin Intervention on Arterial Retention of Remnant Lipoproteins

**Figure 9.**
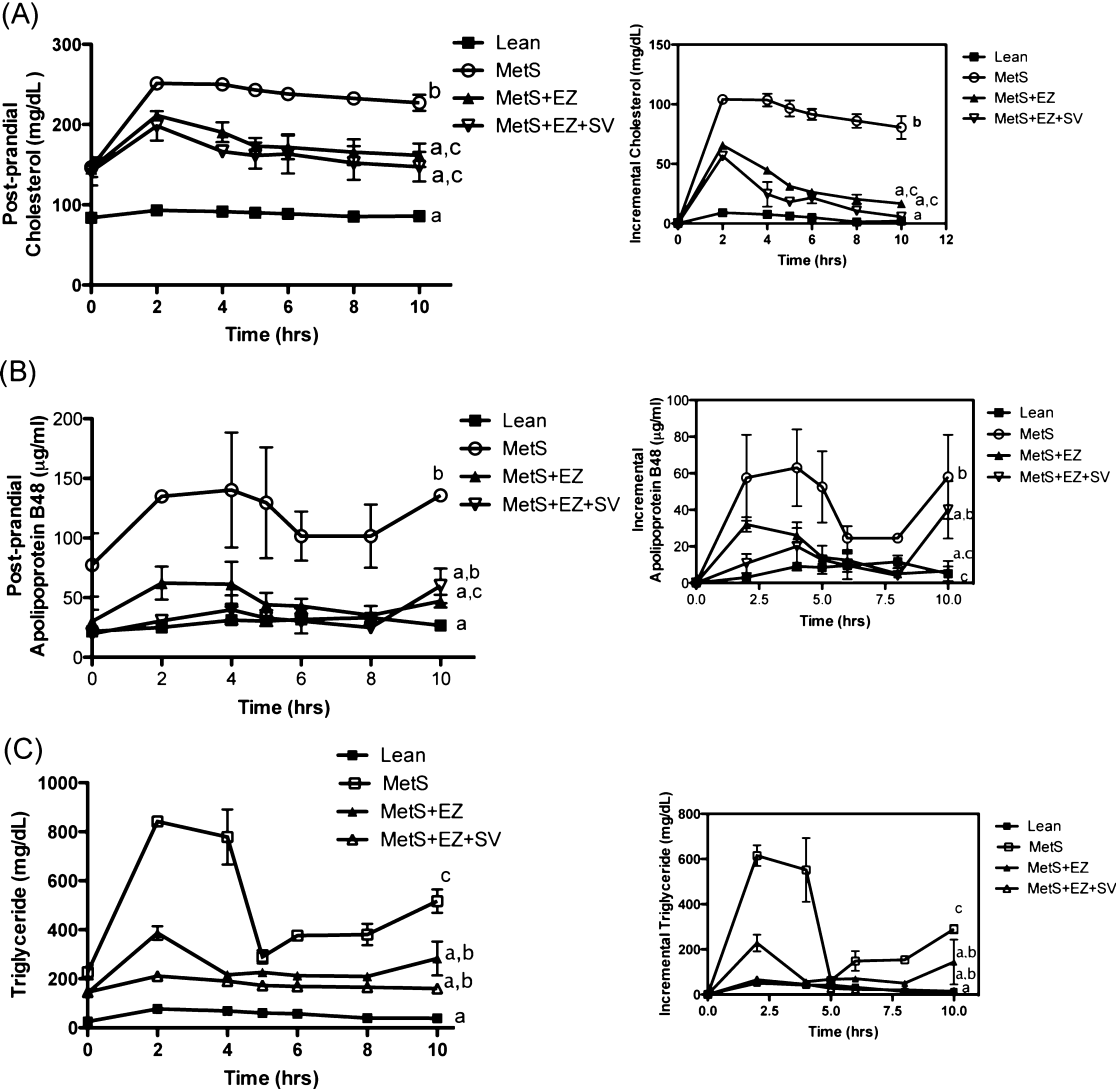
Postprandial (nonfasting) (area under the curve) plasma response for (A) cholesterol, (B) apoB48, and (C) triglyceride after an oral fat challenge (n=4) for lean, MetS, and MetS rats treated with ezetimibe (EZ) alone and in combination with simvastatin (SV). Different letter subscripts (a through c) all indicate a significant difference of *P*<0.05 between the groups according to 1-way ANOVA. MetS indicates metabolic syndrome. Values with the same letter subscript are not statistically different.

Food intake and body weight were not significantly altered by ezetimibe or ezetimibe+simvastatin treatment as compared to MetS control rats (data not shown). In MetS rats, treatment with ezetimibe as well as with ezetimibe+simvastatin significantly reduced the plasma total cholesterol concentration (Table 4), postprandial plasma apoB48 (−60% and −68%, respectively), and postprandial lipid response for cholesterol (Figure 9) relative to untreated MetS rats.

**Table 4.** Fasting Plasma Biochemical Parameters of Lean Control, MetS Rats, and MetS Rats Treated With Ezetimibe Alone or in Combination With Simvastatin for 8 Weeks

Parameters	Lean	MetS	MetS+Ezetimibe	MetS+Ezetimibe+Simvastatin
Cholesterol, mg/mL	0.83±0.05^a^	1.82±0.05^b^	1.45±0.005^c^	1.36±0.08^c^
LDL, mg/dL	16.18±1.9^a^	34.4±3.6^b^	14.9±2.87^a^	15.68±3^a^
HDL, mg/dL	18.85±1.47^a^	49.38±4.98^b^	39.41±2.95^b^	35.21±2.73^b^

Different letter subscripts (a through c) indicate a significant difference of *P*<0.05 between the groups according to 1-way ANOVA. Values with the same letter subscript are not statistically different.

**Figure 10.**
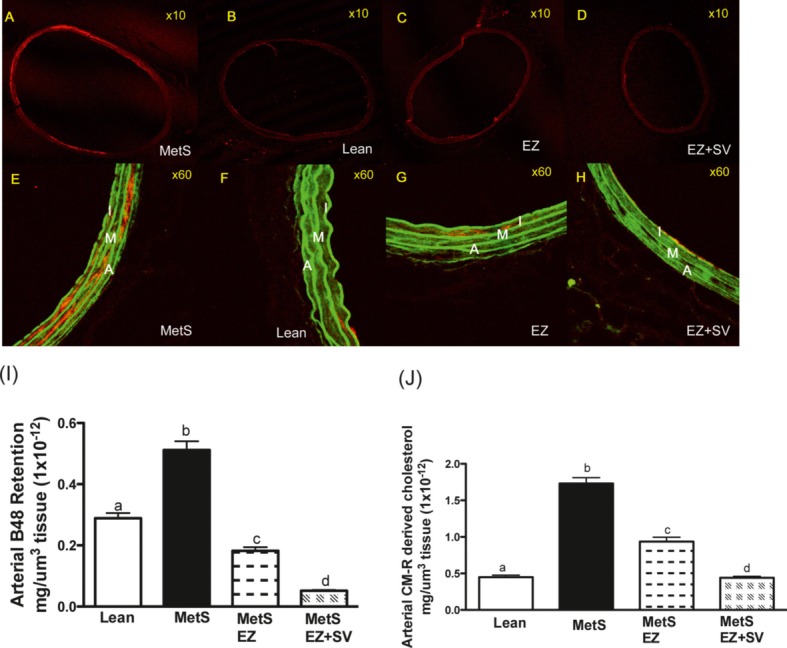
Arterial retention of chylomicron remnants in MetS rats treated with ezetimibe (EZ) alone or in combination with simvastatin (EZ+SV). JCR:LA-*cp* rats were fed a lipid-balanced diet for 8 weeks. MetS JCR:LA-*cp* rats were randomized to a lipid-balanced diet with or without EZ alone (EZ; 0.01% w/w) or in combination with SV (EZ+SV; 0.01+0.01% w/w) for 8 weeks. Images of the arterial retention of intestinal-derived remnant lipoproteins in all treatment groups are shown in A through H. A through D, Fluorescence associated with Cy5-labeled intestinal-derived remnant lipoproteins (red). E through H, Composite overlay of fluorescence associated with remnant lipoproteins (red) and vessel morphology (green). Tissue sections were fixed and fluorescence visualized by confocal laser scanning microscopy (magnifications ×10 and ×60). I indicates intima; M, media; AV, adventitia; and MetS, metabolic syndrome. These data were subsequently quantified (Methods) and presented as the arterial retention of apoB48 (mg/µm^3^ tissue (1×10^−12^) (panel I); and remnants-derived cholesterol (mg/µm^3^ tissue (1×10^−12^) (panel J); (n=3, incorporating both carotids, 2 to 3 sections from each vessel, complete cross sections at ×10 magnification from each rat). Different letter subscripts (a through d) all indicate a significant difference of *P*<0.05 between the groups according to 1-way ANOVA. Values with the same letter subscript are not statistically different.

Treatment of MetS rats with ezetimibe significantly reduced both the arterial retention of remnant particles (−64%) and the corresponding mass of cholesterol (−46%) as compared to untreated MetS rats (Figure 10). As described before, the experimental design for this experiment was autologous, such that remnants were obtained from lean, MetS, ezetimibe-treated, and ezetimibe+simvastatin–treated rats and were perfused ex vivo into similarly treated rats. It was interesting that ezetimibe+simvastatin treatment in MetS rats resulted in an additional reduction in both the number of remnant particles retained in the arterial wall (−72%) and the corresponding mass of cholesterol (−52%) relative to ezetimibe treatment alone (Figure 10I and Figure 10J).

**Figure 11.**
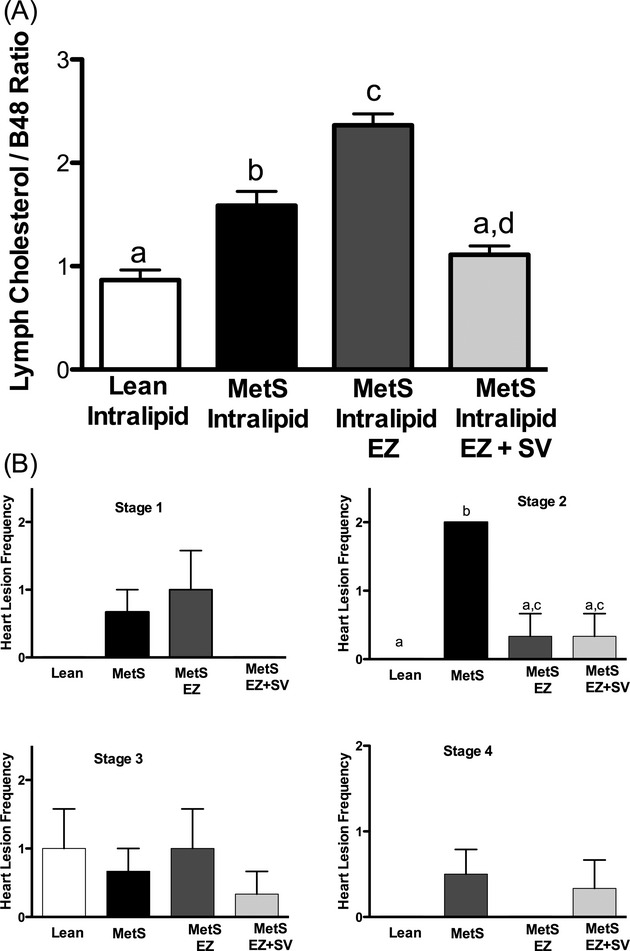
Fed lymphatic cholesterol/B48 ratio (cholesterol per particle) (A) and frequency of myocardial lesions in the hearts (B) of lean and IR rats treated with ezetimibe (EZ) alone or in combination with simvastatin (EZ+SV). Lean control rats were fed a lipid-balanced diet for 8 weeks, whereas MetS rats were randomized to a lipid-balanced diet with or without EZ alone (EZ; 0.01% w/w) or in combination with SV (EZ+SV; 0.01+0.01% w/w) for 8 weeks (A and B). Lymph was collected from the mesenteric lymph duct after intralipid infusion (fed state) (A). Absent bars represent the absence of myocardial lesions in the sections examined (B). Values are mean ± standard error of the mean (n=4), *P*<0.05. Different letter subscripts (a through d) all indicate a significant difference of *P*<0.05 between the groups according to 1-way ANOVA. MetS indicates metabolic syndrome. Values with the same letter subscript are not statistically different.

Additionally, IR rats treated with the combination of ezetimibe+simvastatin had a reduced ratio of lymphatic cholesterol to apoB48 (−52%) relative to untreated controls (Figure 11A).

### Myocardial Lesion Frequency

Hearts from control and treated JCR:LA-*cp* rats exhibited no statistical difference in the frequency of Stage 1, Stage 3, or Stage 4 myocardial lesions (Figure 11B). However, hearts isolated from MetS rats showed a large number of early stage (Stage 2) myocardial lesions as compared to lean control rats (Figure 11B). In addition, hearts isolated from MetS rats treated with ezetimibe alone (−84%; significant at *P*<0.05) and in combination with simvastatin (−84%; significant at *P*<0.05), displayed a marked reduction in the frequency of early Stage 2 myocardial lesions versus control rats (Figure 11B).

## Discussion

### Increased Arterial Retention of Remnant Lipoproteins in MetS

Increased deposition of remnant lipoproteins in MetS rats, as demonstrated by the arterial perfusion (of equivalent particle number) (Figure 1A through 1F), could be due to dyslipoproteinemia or to perturbations in the vessel wall. Crossover perfusion experiments (Figure 2A and 2B) revealed that increased retention of the number of remnant lipoproteins in IR was due to differences in arterial vasculature and was independent of other particle dysfunction per se. It has been demonstrated that type 2 diabetes mellitus can induce compositional differences in LDL (increased apoCIII/apoB molar ratio) and increase the binding of LDL to arterial proteoglycans.^24^ At least in the hyperinsulinemic model studied here, it seems that non–lipid-associated differences in remnant lipoprotein composition do not play a large role in exacerbating particle binding with arterial proteoglycans ex vivo. However, crossover perfusion experiments also demonstrated a further increase in arterial retention, which suggests an exacerbated interaction between remnants from MetS rats and vessels of MetS rats. It is plausible that MetS-induced changes on the remnants significantly increase their susceptibility for proteoglycans with longer chains but not for normal proteoglycans.

When considering the net cholesterol composition of these particles, we found that IR resulted in the overproduction of small, dense, cholesterol-rich particles that caused increased deposition of arterial cholesterol (Figure 3C) in the JCR:LA *cp* rats.

Recently, overproduction of remnant lipoproteins has been shown to contribute to dyslipidemia in subjects with type 2 diabetes mellitus,^8^ and animal studies implicate involvement of several mechanistic pathways.^25,26^ Data from our lymph cannulation studies (Figure 3 and Table 1) confirm that the influence of MetS can increase the rate of secretion and elevate the triglyceride and cholesterol–to–apoB48 ratio.

According to reports from other groups, circulating free fatty acids, microsomal triglyceride transfer protein, monoacylglycerol acyltransferase, diacylglycerol acyltransferase and Niemann-Pick C1-like 1 have been implicated as the cause for chylomicron overproduction in MetS and type 2 diabetes mellitus (reviewed in Williams^27^). We found that MetS rats had significant intestinal hypertrophy (Figures 4 and 5 and Table 2), and this also could contribute to chylomicron overproduction in MetS. Importantly, we found that in MetS, these particles seem to retain their cholesterol-rich composition as a smaller, more atherogenic population of remnant particles as compared to control particles. This also seems to be crucial during the fed state and under conditions of postprandial lipemia (Figures 3 and 6).

### Aortic Remodeling of Biglycan and Decorin in MetS

Several studies have found biglycan and decorin to colocalize with apoB in human atherosclerotic and restenotic lesions.^28^ Recently we have shown that intestinal-derived remnant lipoproteins also colocalize with arterial biglycan in an insulin-deficient model of type 1 diabetes mellitus ex vivo.^12^ McDonald et al^29^ also have reported that a high-fat–fed swine model of type 1 diabetes mellitus had a 4-fold increase in biglycan content in coronary artery lesions. We demonstrate here in the JCR:LA-*cp* rat that in the prediabetic milieu, aortic biglycan protein core content also increases significantly with age and correlates linearly with increasing hyperinsulinemia (Figure 7). We know that the expression of biglycan protein core has been shown to be increased with fatty acids,^30,31^ angiotensin II,^32^ and transforming growth factor-β.^33,34^ Consistent with this, obese JCR:LA-*cp* rats have been shown to have elevated concentrations of transforming growth factor-β^35^ and non-esterified free fatty acids.^36^ In vitro studies with cell culture and smooth muscle cells have demonstrated that transforming growth factor-β can upregulate biglycan expression but can simultaneously downregulate decorin expression.^34,37^

Decorin has been shown to colocalize with apoB in human atherosclerotic specimens^38^ but not in murine atherosclerotic lesions.^39^ Data from our study are consistent with the proposal by Al Haj Zen et al^40^ and do not support the involvement of decorin protein with atherosclerotic risk associated with conditions of IR.

### Effect of MetS on Arterial Sulfated GAGs

We did not observe any age-related changes in total sulfated GAGs between the lean and obese thoracic aortae between phenotypes or with age (Figure 8A). However, Tovar et al^41^ have shown that total sulfated GAG content can increase from birth to 40 years of age in humans, thought to be due to an increase in chondroitin sulfate. The difference between our results and those of Tovar et al might be due to the narrow age range of rats chosen. In our study, the comparative age range of rats would be equivalent to ≍20 to 40 human years of age, and it is interesting that the study by Tovar et al did not report any observable difference in total sulfated GAG content in humans during this same period.

It has been demonstrated that an increase in the length or sulfation of GAG chains can increase the proteoglycan binding affinity for lipoproteins.^28^ Consistent with this, we observed an increase in chain length of arterial GAGs in IR at 12 weeks of age (Figure 8B), which could have contributed further to differences of arterial deposition of remnant particles in our crossover perfusion studies (Figure 2).

Other proteoglycans have been shown to be proatherogenic, including perlecan, which carries heparan sulfate GAGs. However, perlecan has been shown to have conflicting proatherogenic^42,43^ and antiatherogenic effects.^44^ Wasty et al^45^ reported a decrease in heparan sulfate–to–dermatan sulfate ratio in atherosclerotic and type II diabetic arteries. Although we did not measure the perlecan core protein directly in the present study, we did not observe any notable differences in the heparan sulfate chains in our chondroitin ABC lyase–resistant samples (Figure 8B).

### Arterial Retention of Remnant Lipoproteins and Associated Cholesterol Deposition in Response to Ezetimibe and Simvastatin

This study demonstrated that ezetimibe treatment in a model of IR and MetS can ameliorate the deposition of arterial remnants and associated cholesterol ex vivo (Figure 10). It is also intriguing that the addition of simvastatin to ezetimibe seemed to have an additional benefit in reducing arterial cholesterol deposition, which suggests a synergism of independent modes of action. Furthermore, we conclude that decreased cholesterol per chylomicron from rats treated with a combination of ezetimibe+simvastatin (Figure 1A) contributes to decreased arterial cholesterol retention. However, it also has been shown that vascular smooth muscle cells exposed to statins can secrete proteoglycans with reduced binding to LDL,^46^ and this could be an alternative mechanism by which statins can affect arterial remnant retention.

### Frequency of Early-Stage Myocardial Lesions in MetS

Ischemic myocardial lesions constitute a crucial endpoint in CVD. Previous studies with the JCR:LA-*cp* rat strain have demonstrated a correlation between the frequency of myocardial lesions and hyperinsulinemia.^23^ Hearts isolated from MetS rats treated with either ezetimibe (−84%) or ezetimibe+simvastatin (−84%) displayed a significant reduction in the frequency of early Stage 2 myocardial lesions (very recent ischemic lesions undergoing scavenging and repair, characteristic for this strain at this age) versus untreated controls (Figure 1B). The reduction in extravasation, plasma fasting (Table 4) and postprandial lipids (Figure 9), and retention of remnant lipoproteins in the arterial wall (Figure 10) of MetS rats treated with ezetimibe or ezetimibe+simvastatin might explain the reduction in the frequency of early-stage myocardial lesions.

A broader interpretation about “residual risk” should be considered in the context of MetS and type 2 diabetes mellitus. It is now established that the intestine likely upregulates both cholesterol absorption and secretion, contributing to plasma dyslipidemia and CVD development. Once these adaptations are achieved in diabetes mellitus, it is not known whether existing pharmaceutical treatments (eg, statins, fibrates) or a combination of ezetimibe with statins can overcome these factors conclusively. Regardless, it could be important to complement lifestyle changes to the pharmaceutical approach.

Collectively, this study supports the hypothesis that IR and MetS can lead to an acute, exacerbated retention of remnant lipoprotein and associated cholesterol. Furthermore, that increased entrapment of remnant particles could be a result of increased arterial biglycan protein core with longer GAGs or increased propensity of binding by particles during the nonfasted state. We conclude that increased exposure and retention of remnant lipoproteins to arterial proteoglycans in the nonfasted state could in part explain increased progression of atherosclerotic CVD in IR pertinent to conditions of MetS and type 2 diabetes. Furthermore, in addition to the lipid-lowering effects, the combination of ezetimibe and simvastatin synergistically reduced arterial remnant retention.
